# Post-operative Tumor Lysis Syndrome in High-Grade Uterine Sarcoma

**DOI:** 10.7759/cureus.37956

**Published:** 2023-04-21

**Authors:** Hana Qasim, Himil J Mahadevia, Ben Ponvilawan, Hana Hamdan, Anuj Shrestha

**Affiliations:** 1 Internal Medicine, University of Missouri, Kansas City, USA; 2 Internal Medicine, Mayo Clinic, Jacksonville, USA; 3 Pathology, University of Missouri, Kansas City, USA; 4 Hematology and Oncology, University of Missiouri, Kansas City, USA

**Keywords:** spontaneous tumor lysis without precipitating factor, gynec oncology, post-op, tumor-lysis syndrome, uterine sarcoma

## Abstract

Tumor lysis syndrome (TLS) is a well-known oncologic emergency. It is a constellation of metabolic derangements usually observed in hematological malignancies due to rapid cell lysis, typically due to chemotherapy or radiotherapy initiation. Spontaneous TLS is an unusual complication in solid malignancies, and only a few cases have previously been reported for spontaneous TLS in gynecological malignancies. We report a case of TLS in a 50-year-old female patient shortly after resection of high-grade uterine sarcoma. We review previous TLS cases in uterine malignancies and the associated morbidity and mortality.

## Introduction

Tumor lysis syndrome (TLS) is a constellation of metabolic disturbances that occur when many malignant cells undergo lysis within a short period. The effect is the release of various intracellular substances, including uric acid, potassium, and phosphorus, into the extracellular space [1]. This leads to hyperkalemia, the formation of calcium phosphate crystals, subsequent deposition of uric acid and calcium phosphate crystals in the renal tubules, and kidney injury.

Tumor lysis syndrome is a common complication in hematological malignancies, either after starting chemotherapy or spontaneously in high-grade and bulky lymphoproliferative diseases [2]. TLS is much less likely to be seen in solid malignancies and typically occurs after chemotherapy in rapidly growing, high-grade, and metastatic tumors, such as small and non-small cell lung cancer. 

## Case presentation

We present a 50-year premenopausal patient with a past medical history of type 2 diabetes, hypertension, uterine fibroids, and uterine artery embolization for abnormal uterine bleeding. The patient presented to the hospital with complaints of abdominal pain and distension for four days, associated with bloating, nausea, and worsening of her chronic constipation. The pain radiated to her chest and flanks and was worse with movement, and it did not improve with non-steroidal anti-inflammatory drugs or laxatives. She denied any recent abnormal uterine bleeding or abnormal vaginal discharge. Upon presentation, the patient was visibly in discomfort. Her vital signs were as follows: Normothermic - 97.9°F, tachycardic - 122, blood pressure - 109/88 mmHg, respiratory rate - 16/min, and oxygen saturation of 97% on room air. Her examination showed a well-developed and well-nourished middle-aged lady with clear lungs bilaterally. Her abdomen felt distended with diffuse guarding and tenderness to palpation. The general exam showed no palpable enlarged lymph nodes or edema in the lower limbs. The rest of the physical examination was unremarkable.

**Figure 1:**
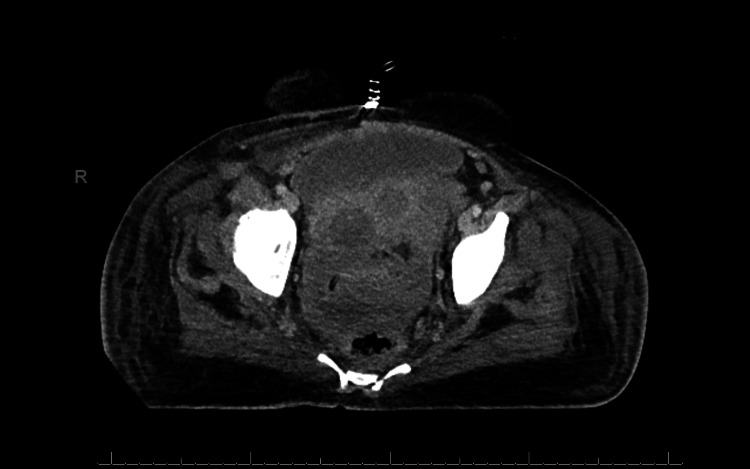
CT scan shows a large Pelvic heterogeneous soft tissue density mass.

Because of her symptoms and the previous history of a large uterine fibroid, a CT abdomen and pelvis was done, which showed a large heterogeneous soft tissue density mass with areas of calcification arising out of the pelvis and extending to the mid-upper abdomen level (Figure 1). There was mild free fluid noted within the lower abdomen and pelvis.

MRI was performed the next day and redemonstrated a large multilobulated soft tissue mass arising from the pelvis and extending into the lower abdomen, for which differential considerations include multifocal uterine leiomyomas with cystic and hemorrhagic degeneration, or leiomyosarcoma, given the large size and the evidence of omental caking. Omental caking is a radiological sign of abnormally thickened greater omentum and frequently indicates advanced peritoneal disease.

For diagnostic and therapeutic reasons, a laparotomy was done, and it revealed a fleshy mass in the cul-de-sac involving the rectosigmoid colon and posterior aspect of the vaginal cul-de-sac. The patient underwent a total abdominal hysterectomy with bilateral salpingectomy, bilateral oophorectomy, and partial omentectomy. She was clinically stable till two weeks after surgery, when she had worsening fatigue and drowsiness. Lab tests showed a worsening metabolic panel with a rise in her creatinine, phosphorus, and potassium levels and a drop in her calcium level (table 1).

**Table 1:** Comparison between patient's blood tests on admission and 2 weeks after the surgery. BUN: Blood urea nitrogen; LDH: lactate dehydrogenase

Blood test	On admission	2 weeks after the surgery	Normal value
Creatinine	0.48 mg/dl	1.8 mg/dl	0.59 to 1.04 mg/dL
Potassium	3.1 mmol/L	5.2 mmol/L	3.6 to 5.2 mmol/L
Phosphorus	3.9 mg/dl	7.3 mg/dl	2.8 to 4.5 mg/dL
BUN	16 mg/dl	72 mg/dl	6 to 24 mg/dL
LDH	No baseline	381 IU/L	105 to 330 IU/L
Uric acid	No baseline	15.8 mg/dl	1.5 to 6.0 mg/dl
Calcium	9.4 mg/dl	7.3 mg/dl	8.5 to 10.2 mg/dL

The above findings are indicative of tumor lysis syndrome. Most likely precipitated by the recent abdominopelvic surgery. The patient had deterioration in her clinical status, evident by hypoxemic respiratory failure and hypotension. She transferred to the critical care unit for closer observation. TLS was managed with IV fluids resuscitation, Rasburicase 3 mg once, and phosphate binders. The patient's condition improved without hemodialysis, and she was transferred back to the medical floor after three days. 

**Figure 2:**
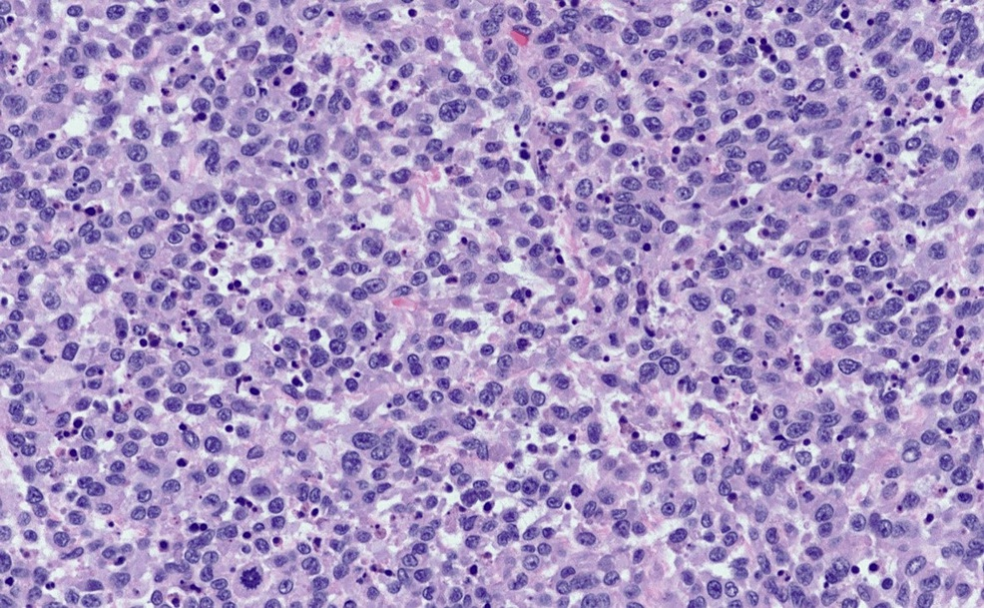
High-grade uterine sarcoma – High-grade endometrial stromal sarcoma (HG ESS) vs. leiomyosarcoma (ULMS)

The final pathology report (Figure 2) was consistent with high-grade uterine sarcoma-high-grade endometrial stromal sarcoma (HG ESS) versus leiomyosarcoma (ULMS). Based on imaging and operating findings, it was labeled a stage IV uterine sarcoma. She started on gemcitabine and docetaxel chemotherapy in the hospital. She tolerated the chemotherapy with no immediate adverse events. However, she remained deconditioned, had poor oral intake and severe abdominal pain requiring IV opioids, and thus had a prolonged hospital stay. After the second cycle of chemotherapy, a CT scan of the abdomen and pelvis was done, and it showed disease progression. The care goals were discussed with the patient and family members, and she opted for comfort-based care with the hospice care team.

## Discussion

Tumor lysis syndrome (TLS) is a well-known oncologic emergency. It is a constellation of metabolic derangements commonly observed in high-grade hematological malignancies [1], especially in high-grade B-cell lymphomas, such as Burkitt lymphoma and acute lymphocytic leukemia. It usually happens after the initiation of treatment for malignancies that are highly sensitive to chemotherapy. The death and lysis of many malignant cells, followed by the release of intracellular substances, lead to the typical findings of hyperkalemia, hyperphosphatemia, hypocalcemia, hyperuricemia, and acute kidney injury. Kidney impairment is mainly due to uric acid nephropathy and partly due to other factors like hypocalcemia. Despite the efforts for prevention and early detection of TLS, morbidity and mortality remain high. They are mainly related to the complications of acute kidney injury, metabolic acidosis, and electrolyte disturbance-related cardiac arrhythmias. Tumor lysis syndrome can present in hematological malignancies, Especially Burkitt's lymphoma, high-grade diffuse large B-cell lymphoma, and acute lymphocytic leukemia. However, it mainly occurs as a post-chemotherapy complication in tumors susceptible to chemotherapy [2]. Prophylactic treatment with hydration, allopurinol, or Rasburicase is often indicated before treatment initiation [3]. 

TLS is much less likely to occur in solid malignancies after chemotherapy initiation. Spontaneous TLS is a rarely observed complication in solid malignancies, which might contribute to late diagnosis when it happens. Nevertheless, it can be observed in high-grade malignancies, high tumor burdens, and other precipitating factors, such as dehydration and renal impairment. There are only a few cases of reported TLS in gynecological malignancies. Most reported cases are of ovarian carcinoma following cytotoxic chemotherapy [4]

Upon literature review, we found few reported cases of TLS in uterine carcinomas and sarcomas, which happened either post-chemotherapy, post-radiotherapy, or spontaneously (Table 2). The unifying character of all these patients was having an advanced, bulky disease with metastases, which makes it very clear that such patients need prompt observation following any kind of intervention, including chemotherapy, radiotherapy, surgery, or even for patients who do not receive treatment because of medical reasons or patient choice. Post-operative TLS was rarely reported in solid malignancies such as colon cancer [5].

**Table 2:** Overview of tumor lysis syndrome cases in uterine malignancies.

Type of malignancy	Age	Stage	Precipitating factor	Outcome
Recurrent endometrial cancer [6]	63 yrs	Metastatic	Chemotherapy	Death
Uterine epithelioid leiomyosarcoma [7]	36 yrs	Metastatic	Chemotherapy	Recovery
Clear cell carcinoma tumor [8]	85 yrs	metastatic	Radiotherapy	Dialysis, death
Endometrial adenocarcinoma [9]	33 yrs	Metastatic	Spontaneous	Recovery
Uterine Leiomyosarcoma [10]	58yrs	Metastatic	Spontaneous	Death
Endometrial adenocarcinoma [11]	75 yrs	Metastatic	Spontaneous	Death
Uterine high-grade serous carcinoma and minor component (10%) of high-grade sarcoma [11]	61 yrs	Metastatic	Chemotherapy	ICU admission, dialysis

Reviewing the literature, we found that only two of seven patients fully recovered after developing TLS without significant morbidity. We believe that high morbidity and mortality in TLS in solid malignancy are attributed to tumor lysis syndrome developing in patients with metastatic disease with poor general health conditions and limited treatment options.

## Conclusions

TLS is a rare but life-threatening complication in uterine malignancy, especially in bulky and metastatic cases. The metabolic panel should be followed closely after starting any therapeutic model, including chemotherapy, radiotherapy, and surgery. Upon diagnosis of TLS, patients should be managed promptly in high-dependency units with close observation.
